# Evaluating Mobile Survey Tools (MSTs) for Field-Level Monitoring and Data Collection: Development of a Novel Evaluation Framework, and Application to MSTs for Rural Water and Sanitation Monitoring

**DOI:** 10.3390/ijerph13090840

**Published:** 2016-08-23

**Authors:** Michael B. Fisher, Benjamin H. Mann, Ryan D. Cronk, Katherine F. Shields, Tori L. Klug, Rohit Ramaswamy

**Affiliations:** 1 The Water Institute at UNC, Department of Environmental Sciences and Engineering, University of North Carolina at Chapel Hill, Chapel Hill, NC 27599, USA; rcronk@live.unc.edu (R.D.C.); kshields@email.unc.edu (K.F.S.); tori.klug@gmail.com (T.L.K.); 2 DAI, 7600 Wisconsin Avenue, Bethesda, MD 20814, USA; benjamin.h.mann@gmail.com; 3 Public Health Leadership Program, Gillings School of Global Public Health, University of North Carolina, Chapel Hill, NC 27599, USA

**Keywords:** water, survey, mobile, data, ICT, evaluation, information, communications, technology, software

## Abstract

Information and communications technologies (ICTs) such as mobile survey tools (MSTs) can facilitate field-level data collection to drive improvements in national and international development programs. MSTs allow users to gather and transmit field data in real time, standardize data storage and management, automate routine analyses, and visualize data. Dozens of diverse MST options are available, and users may struggle to select suitable options. We developed a systematic MST Evaluation Framework (EF), based on International Organization for Standardization/International Electrotechnical Commission (ISO/IEC) software quality modeling standards, to objectively assess MSTs and assist program implementers in identifying suitable MST options. The EF is applicable to MSTs for a broad variety of applications. We also conducted an MST user survey to elucidate needs and priorities of current MST users. Finally, the EF was used to assess seven MSTs currently used for water and sanitation monitoring, as a validation exercise. The results suggest that the EF is a promising method for evaluating MSTs.

## 1. Introduction

### 1.1. Background

Access to mobile phones is growing rapidly, with over 7 billion connections worldwide [1], and over 800 million in sub-Saharan Africa alone, the world’s fastest-growing market, with smartphone connections expected to exceed 500 million by 2020 [2]. The widespread availability and decreasing cost of mobile devices make mobile data collection an attractive option for many programs. Information and communications technologies (ICTs) such as smartphones, tablets, basic phones, and other mobile devices, in combination with wireless networks and software programs and applications for field data collection, can comprise useful mobile survey tools (MSTs) for collecting, aggregating, and analyzing field-level data to improve the effectiveness of many types of operations, including national and international development work [1,2,3,4,5,6] in general, and water, sanitation, and hygiene (WaSH) programs [7,8,9,10,11,12,13,14,15] in particular. Such data can aid in more effective decision-making to enhance program impact. In the context of mobile data collection, an MST can be defined as the integration of four components; (1) mobile hardware for data entry; (2) data collection software (e.g., a mobile data collection application, or “app”); (3) a mobile network connection that allows data transmission to a remote server; and (4) data aggregation and analysis (e.g., through an online dashboard) [3]. For the purposes of this paper, when comparing and evaluating MSTs, we will consider only the second and fourth components (data collection app and data aggregation and analysis platform), as these are typically the elements supplied by various MST developers, while hardware and network options tend to be sourced separately and vary from one deployment to the next. Furthermore, while the category of MSTs is a subset of the broad category of ICTs, which can include everything from personal computers to telephones and software of all kinds, the term “ICT” is often used colloquially in the context of field data collection to refer to MSTs. We will use the term MST for the purposes of this discussion, but have used the more colloquially familiar “ICT” in user surveys because this term is more broadly recognized.

Many MSTs for field data collection are now available, with a variety of features and pricing schemes, ranging from free tools to institutional plans costing thousands of dollars per year. Such tools have found widespread use in international and national development sectors, with health [16,17,18], agriculture [3,19,20], and microfinance [21] applications, and are now being employed in the WaSH sector as well. WaSH applications have included water point mapping [9,12], as well as tracking water point functionality and service levels [10,13,14,15], and in some cases have led to substantial adjustments in reported service and coverage levels [5]. Furthermore, MSTs can make higher quantities and quality of data available to a variety of stakeholders far more rapidly than has been the case with paper-based tools [6,7,11], can integrate GPS coordinates, images, and other data types with text and numerical survey data, and can often pay for themselves through gains in operational efficiency [5]. MSTs can also integrate automated quality assurance/quality control features, such as the use of auditing functions [22] and algorithms to identify fabricated data [23,24]. Numerous MST options are currently available: a quick search found over 30 free or paid (i.e., commercially available) MSTs online, and many others may exist (Google search terms included: “mobile survey or survey app or ICT”). Thus, a wide variety of MST options and features is available to organizations collecting field-data.

While prior studies have suggested general criteria for selecting an MST, such as suitability of features to data collection needs and ability to design forms consistent with data collection needs [3], these approaches have largely been limited to suggesting factors that users should contemplate when selecting an MST. While these contributions have been valuable in shaping discussions around MSTs, there remains a need for an implementable framework that can be used to systematically compare and evaluate MSTs.

### 1.2. Study Objectives

The purpose of this work is to develop a systematic framework for evaluating MSTs for field-level monitoring across a wide variety of applications. The primary goals of this study are:

1. To survey current user practices and attitudes around MSTs for illustrative purposes.
2. To develop a rigorous, systematic, and implementable evaluation framework, informed by user surveys and existing software evaluation standards, to compare MSTs across multiple quality and performance metrics.
3. To validate the proposed framework by providing objective assessments of several MSTs used for WASH monitoring and evaluation.

## 2. Materials and Methods

In order to support the above objectives, we created and administered a survey of MST users’ experiences, preferences, priorities, and evaluations of their current MST. We then used the results from this survey, as well as existing frameworks for evaluating software quality, to construct a systematic evaluation framework for MSTs. Finally, we validated this framework by using it to compare a small sample of MSTs currently in use within the water, sanitation, and hygiene (WaSH) sector (a field with which we happen to be particularly familiar).

### 2.1. Online Survey of MST Users

An online survey was developed to determine the rates at which different MSTs were used for WaSH data collection in the field, to identify key strengths and weaknesses of these MSTs, and to examine users’ levels of satisfaction with and reasons for selecting the MSTs that they currently use. The MST user survey provided necessary inputs for the creation of the evaluation framework, such as critical features identified by practitioners, and also identified MSTs commonly used by survey respondents, informing the selection of test cases for the evaluation framework.

A 16-item questionnaire was developed using the SurveyMonkey online survey platform (Figure S1). This questionnaire was piloted among a group of approximately 40 graduate students and staff at the University of North Carolina, then sent to approximately 900 members of the Rural Water Supply Network’s water point mapping “D-group” e-mail listserve (a group that includes many users of MSTs, as well as individuals interested in MSTS and waterpoint mapping) and was also disseminated via direct emails to WaSH implementers receiving funding from the Conrad N. Hilton Foundation, and shared openly via personal social media outlets of the authors (i.e., Twitter and LinkedIn). The audience reached by these channels is varied, but comprises WaSH and development researchers and practitioners in universities, NGOs, international and bilateral aid agencies, government agencies, and other individuals with interests in WaSH, technology, and/or development. The total estimated number of recipients reached by these channels was approximately 1000 and comprised a convenience sample. The questionnaire included free-response text and numeric fields for questions about institutional affiliations, as well as standard Likert scale response options for questions about user satisfaction and the ease/difficulty of using various functions. Finally, free-response fields were also used for open-ended questions about the various advantages and drawbacks of users’ current MSTs. The survey protocol was submitted to the Institutional Review Board at the University of North Carolina at Chapel Hill, which determined that the online survey does not constitute human subjects research as defined under federal regulations (45 CFR 46.102 (d or f) and 21 CFR 56.102(c)(e)(l)) and does not require IRB approval (Study #: 13-1292).

#### Inclusion Criteria

Forty respondents provided answers to survey questions (corresponding to a response rate of approximately 4%). Of these, 31 provided relevant responses to free-response questions, while nine provided responses to free-response questions that were deemed irrelevant. Free responses were deemed irrelevant if they were unrelated to MSTs (e.g., unrelated comments on local politics), if they were from non-MST users (e.g., organizations using pen and paper for data collection), or if they were related to MSTs but unrelated to the questions posed (e.g., a complaint about mobile phone battery life in response to a question about the types of activities for which the respondent’s organization uses MSTs). Those respondents who provided more than 50% irrelevant responses to free-response questions, or who provided responses indicating that their organization had never worked with MSTs, were dropped from the survey. Relevance was assessed by the researchers using the following criteria: (1) Could the response provided reasonably be interpreted as addressing the question posed? and (2) Could the response reasonably be interpreted as coming from a respondent who has used an MST at least once? Surveys were assessed by three reviewers, and those for which ≥50% of the responses were deemed irrelevant by ≥2 reviewers were dropped. Where responses were potentially relevant but unclear due to grammatical errors, they were included and interpreted to the best of the researchers’ abilities.

### 2.2. Development of the Evaluation Framework

Within the information and communication technologies for development (ICT4D) subsector, many user groups and researchers have identified a knowledge gap in evaluating and comparing MSTs of a similar nature. To address this gap, a framework was developed for evaluating individual tools and providing standardized methods which would allow comparative analysis of all such MSTs. The resulting systematic Evaluation Framework (EF) consists of qualitative and quantitative metrics to provide a summary analysis of any given MST.

The EF was designed using International Organization for Standardization (ISO) recommendations for quality modeling of software and web-based tools. ISO defines a quality model (QM) as a “defined set of characteristics, and of relationships between them, which provides a framework for specifying quality requirements and evaluating quality”. QM frameworks have long been used for traditional websites; however, the application of such standards to mobile applications is in its infancy. Quality models often are targeted at developers or managers of software systems; however, EF also incorporates the needs of end users and is designed to be accessible to technical and non-technical experts.

ISO standards ISO/IEC 25010 [25] and ISO 9126 [26] were consulted and adapted to develop the quality model for the EF. The QM consists of 11 core characteristics and 33 sub-characteristics (Table 1). Briefly, EF includes metrics of functionality, reliability, usability, efficiency, maintainability, and portability. Functionality metrics include both measures of functional adequacy (i.e., are the required features available?) and functional correctness (i.e., do these features function correctly during testing?). Reliability metrics primarily assess the observed stability of MST components during testing. Usability metrics assess both the ease of use of various features and the level of training required for a typical new user to become proficient. Maintainability metrics primarily assess the visibility and modifiability of MST code and functions. Portability metrics primarily assess the platforms on which each MST operates, and the ease of installation and adaptability of each tool. Efficiency metrics assess the speed with which the app and dashboard components of each tool can be deployed and used under simulated test conditions, as well as the resource utilization of each tool. The framework also includes quality-in-use metrics, including performance during testing, user satisfaction, and freedom from risk (e.g., risk of data loss), as well as user perceptions with respect to the learnability of the tools. Finally, the relative cost (if any) and the flexibility of each MST were considered.

The characteristics and sub-characteristics were mapped to the EF through a 75-point MST evaluation questionnaire (MEQ) to assess each MST and generate a score or result for each question (Table S1). Four testers were recruited from among graduate students and researchers at The University of North Carolina at Chapel Hill (UNC) to assess each MST using the MEQ. The MEQ contained two types of questions: “individual questions,” such as performance ratings (e.g., ease of use of the MST’s online dashboard), for which each tester assigned an individual score to the MSTs tested, and “objective questions,” such as objective MST characteristics (e.g., available features or operating systems supported), which were entered only once by a single tester. The MEQ was broken into sections to evaluate each tool over the entire Data Management Value Chain to capture points of interaction between characteristics, sub-characteristics, and the operation of the tools (Figure S2).

### 2.3. Evaluation of Tools Using the Framework

Based on the results of the online survey of MST users, the two MSTs mentioned by more than one respondent each (FLOW and ODK) were selected for testing. Five additional MSTs were added to the list based on a review of existing MSTs used for WaSH monitoring by one or more national governments, major international or bilateral development organizations, or major international companies. Sources for identifying these additional MSTs included the literature [7,11], a shared database developed by members of the RWSN D-group listserve [27], personal communications with colleagues, and the websites of major international WaSH implementers. The resulting set of seven WaSH MSTs included: Akvo FLOW (Akvo Foundation, Amsterdam, The Netherlands); Open Data Kit (ODK; open source); Magpi (Magpi, Washington, DC, USA); iFormbuilder (Zerion Software, Herndon, VA, USA); Fulcrum (Spatial Networks, Inc., St. Petersburg, FL, USA); mWater (mWater, New York, NY, USA); and Survey CTO (Dobility, Inc., Cambridge, MA, USA). Numerous other MSTs are also available. This work was limited to the current subset in order to demonstrate the application of the EF and its potential to differentiate among MSTs with respect to performance and characteristics without incurring the substantial costs associated with a more exhaustive analysis.

In order to evaluate these MSTs using the EF, all testers completed the individual questions in the MEQ for each MST chosen, and a single tester (in this case, the primary author) completed the objective questions. As part of this evaluation, a customized standard test questionnaire (STQ, Table S2) was developed by the authors, containing questions relevant to the MST application selected for this case study: monitoring and evaluation of WaSH programs. Each tester recreated the STQ using the dashboard of each of the seven demonstration MSTs, and then completed the STQ during a simulated data collection session using the MST’s mobile app. A variety of hardware options and operating systems were used for these evaluations. The performance of the dashboard and app with respect to these tasks, as well as the time required to complete each step and the estimated amount of training required for a typical user to become proficient in each step, were reported by each tester. In order to avoid biasing the survey completion times of those MSTs which lacked some of the features necessary to complete the standard evaluation survey, a correction of +10 s (the average time to complete a question in the STQ) was added to the survey completion time for each question that could not be successfully completed due to missing features. A correction factor of +70 s was added to the survey creation time for each question that could not be generated, by the same rationale.

## 3. Results

### 3.1. User Survey

A total of 40 responses were received from a wide variety of organizations with staff sizes ranging from two to 700 (mean = 106). These organizations represented international and local non-governmental organizations (NGOs), universities, bilateral aid organizations, local and multinational private enterprises, and government agencies. Surveys were received from 14 countries.

From these 40 respondents, 31 relevant responses were collected; 28 specifically listed WaSH MSTs used by their organization, while three others described their user experiences with proprietary MSTs but did not provide the names of these tools. The most widely used MST was Open Data Kit (ODK), used by nine respondents, and a 10th mentioned that ODK was used by many of their member organizations. Seven reported using their own proprietary or custom MST, while four reported using Akvo FLOW, and FLOW use was mentioned by two additional users who responded “Other” (Table 2).

Respondents reported using MSTs for a number of applications (Table 3, Table S3). The most commonly listed were waterpoint data collection (75%), community surveys (66%), household surveys (59%), waterpoint mapping (56%), and field activity reporting (56%).

Respondents listed a variety of reasons for selecting their current MST (Table 4). Ease of data input was the most commonly cited factor (61%), followed by ability to export data in desired format (52%), cost (48%), and ease of survey creation (42%).

#### User Satisfaction

Users reported high levels of satisfaction with the field data collection capabilities of their MSTs, although satisfaction with MST dashboards was lower (Table 5). Ninety percent of respondents reported that they would recommend their current MST to other users. Among users of different MSTs, those using ODK and FLOW reported their satisfaction with the mobile app at 8.6/10 and 8.3/10, respectively, and their satisfaction with the dashboard at 4.3/10 and 7.0/10, respectively (Table 6). There were not a sufficient number of responses from users of other MSTs for user satisfaction levels to be reported for other individual MSTs; these data were therefore aggregated into two additional categories, “Proprietary” and “Other”.

Respondents listed a variety of mobile app features as being beneficial to their work. Most commonly cited were ease of use and GPS features (Table 7). Many respondents also listed features of the individual mobile platforms, devices, and mobile networks that they were using to run MST tools, suggesting a lack of differentiation between mobile device hardware, native software and other applications, mobile network connectivity, and the MST software package being used. Other responses, such as battery life, reflect performance variables that are functions both of the mobile device being used, the MST software in question, and the other software operating in the background.

When asked why these features were beneficial, the most common reason that respondents cited was that these features made the MST easier to use, accelerated data collection, facilitated analysis and dissemination, accelerated information transfer from the field to a central database, and improved data quality. Respondents listed a number of MST mobile app features that were problematic. The most commonly listed feature, network connectivity, was a characteristic of mobile network coverage, rather than the software components of the MST used, and was omitted along with other issues unrelated to MST software design and performance. The most frequently cited MST-specific problems were related to the performance of GPS features and to the MST’s limited output and reporting options (Table 8). Users also cited shortcomings that were not specific to a particular feature of the MST, such as data loss or lack of availability on their preferred mobile device platform. All those listing data loss as a problem were ODK users.

Those users who elaborated on the reasons that the identified features were problematic reported that these limitations caused delays in the field, resulted in data loss, or complicated the use of the MST by field staff without strong information technology (IT) backgrounds.

Respondents listed a limited number of dashboard features as being beneficial to their work. Most commonly cite were ease of use and export features (Table 9). Many respondents also reported that they had not used the dashboard features of their MST. Respondents also listed a number of MST dashboard features that were problematic. Reporting features were the most commonly listed source of difficulties, followed by poor performance over slow network connections (Table 10). 

Those users who elaborated on the reasons that the identified features were problematic reported that these limitations made it difficult for users without specialized IT knowledge to use the dashboard, and prevented the use of data for all desired purposes.

Overall, 88% of respondents said they would recommend their current MST, citing ease of data collection, improved data quality, and improved project outcomes due to increased monitoring efficiency. These results were used to inform the development of the evaluation framework. Specifically, the MEQ was developed in such a way as to ensure that the MST characteristics most important to survey respondents, including ease of use, cost, speed, and learning curve, were well represented in the evaluation questions.

### 3.2. Evaluation Framework

#### 3.2.1. Evaluation Protocol

Seven MSTs were evaluated: Akvo FLOW, OKD, Magpi, iFormbuilder, Fulcrum, mWater, and Survey CTO. These MSTs were tested by the authors on a range of platforms and mobile devices, and the dashboards were tested on PCs running Windows 7 and Macintosh computers running Mac OS X. Evaluations were conducted in 2014 and 2015.

#### 3.2.2. Cost

The most significant difference among MSTs in terms of general characteristics was cost: ODK and mWater are free to use, while the other five MSTs have costs between $100 and $10,000/yr depending upon usage rates (Table 11). In the case of the free MSTs, ODK is an open-source development project supported through grants and donations, and maintained by researchers at the University of Washington and volunteer developers; mWater offers basic functionality for free, and charges institutional clients to develop new features and functionality (which are then made freely available to all users). The merit and sustainability of the various MST business models are beyond the scope of this work.

#### 3.2.3. Performance and Features

MSTs were also evaluated based on the features they provided and the performance of those features when the MSTs were installed on test devices and deployed in the laboratory and the field. Of the seven MST apps tested, three possessed all the features required to complete the full STQ (test survey). The remaining MST apps were missing one to two of the features required to complete the STQ form, typically the ability to scan barcodes and/or record video (Table 12). For two of the seven MST apps, all available app features performed correctly during tests. However, for the remaining MSTs tested, one or more app features was found to perform incorrectly in one or more separate tests. Two of the apps tested crashed (once each) during the completion of the STQ by four testers. All of the apps tested functioned offline. Of the seven dashboards tested, five performed without any problems (except for the missing features noted above); however two did not support skip logic that was adequate to create the complete STQ (i.e., supporting multiple dependencies and dependencies on numeric responses). None of the dashboards tested functioned offline.

#### 3.2.4. Ease of Use and Learning Curve

The ease of use of each MST’s dashboard and app were assessed by each tester during the testing procedure on a Likert scale ranging from 1 (little difficulty) to 5 (very difficult), and the results were averaged across all testers. An overall ease-of-use score was constructed by summing the individual ease-of-use scores across all tasks assessed (app installation, configuration, and navigation; account setup; dashboard navigation; form construction). Fulcrum was found to be the easiest MST to use, followed by mWater, and then a close grouping of iFormbuilder, Magpi, and FLOW (Table 13). The greatest difficulties were experienced with app configuration and form construction.

The learning curve was defined as the estimated amount of training that a typical user would require to be able to competently operate the app and dashboard. This was estimated by each tester on a scale ranging from minutes to one full day or more, and the results were averaged. mWater and Fulcrum were found to have the quickest MST dashboards to master, followed by iFormbuilder, Magpi, and FLOW, with identical estimated learning curves (Table 14).

#### 3.2.5. Speed

The speed of use of each MST was assessed by timing the creation and completion of the standard survey form by each tester. These times were adjusted for the number of survey questions that could be correctly created and completed. Survey completion times varied little between different MSTs, while survey creation times varied more substantially. Overall, Fulcrum and iFormbuilder were the fastest, followed by ODK, mWater, and FLOW (Table 15).

#### 3.2.6. Overall Rank

An overall rank was assigned to each MST based on its (equal-weighted) rankings for cost, ease of use, performance, learning curve, and speed. Fulcrum was found to be the top-ranked MST, based on this formula, followed by mWater and iFormbuilder. Magpi, FLOW, Survey CTO, and ODK were all clustered closely together for ranks 4–7 based on this ranking scheme (Table 16).

## 4. Discussion

### 4.1. Discussion of Results

This work reviews some of the key features and functionalities demanded by MST users in the WaSH sector, and describes the development and application of a systematic evaluation framework for assessing the relative performance and suitability of seven MSTs with respect to their use for mobile data collection in WaSH. The evaluation framework developed in this study is based on international standards for the evaluation of software quality, adapted to the specific case of MST assessment using information gathered from the MST literature and an online user survey. This framework was further refined through application to the evaluation of seven MSTs tools currently used in the WaSH sector. As a result, the authors believe that this evaluation framework represents the first rigorously designed and tested framework for the evaluation of MSTs for field-level data collection.

A review of user preferences revealed a strong focus on cost, speed, ease of use, and fast learning curves. Respondents were often unable to distinguish between features of the MST software and those of the hardware and mobile network they were using, but required all these systems to function in such a way as to facilitate rapid and easy data collection with minimal training requirements and minimal risk of lost data. Users primarily reported using MSTs for waterpoint mapping, suggesting that integration of mapping with survey-based data collection may still be in its infancy among WaSH MST users. Overall, most MST users reported very high satisfaction with their current MST tool, regardless of its features. This tends to suggest that the majority of WaSH MSTs in use are functionally adequate for many users’ current applications, but that “word-of-mouth” may not be a useful method for selecting the best MST options available, as willingness to recommend an MST did not seem to be particularly sensitive to its features, among our survey sample. These results may also suggest that most users find MSTs to be far superior to the paper-based data collection tools they may have used previously, regardless of the features and characteristics of the MST they use now. We may therefore speculate that while MSTs differ from each other with respect to features and performance, these differences may be small in comparison to the substantive advantages of MSTs in general vs. pen-and-paper tools.

One of the most important criteria identified by survey respondents for selecting MSTs, and one of the criteria for which the most variability was observed in this work, was cost. The aggregate performance of the five “paid” MSTs was not significantly better than the aggregate performance of the two free MSTs tested, when cost was removed from the overall composite score. This suggests that at the time of this study, differences in performance between paid and free MSTs may be small compared to differences in cost (95% confidence interval). Furthermore, performance did not vary monotonically with cost, suggesting that costlier MSTs did not always deliver greater value. Most of the MSTs studied offered broadly comparable levels of data security and instructional materials, irrespective of cost. The quality of technical support available for free vs. paid MSTs was not directly assessed in this study; however, it should be noted that ODK (one of the two free MSTs) offers support primarily through an online user community, while other MST developers (paid and free) offer support directly, in addition to any user communities they may have.

The systematic evaluation of the seven MSTs selected using the newly created evaluation framework also indicated that while most of these MSTs were adequate for the creation and completion of the case study test questionnaire (STQ), some tools lacked key question types (i.e., barcode and video) and skip logic features (i.e., multiple dependencies and dependencies on numeric data) needed to construct and complete the full STQ. Substantive differences also emerged with respect to the performance, ease of use, cost, speed of use, and learning curves of the various options. A combined ranking of evaluation results across these categories revealed that one of the MSTs tested performed far better than the others according to the current evaluation framework.

The evaluation framework indicated important differences between the MSTs tested, even though all seven of the MSTs tested appeared to be broadly suitable for most field-level monitoring applications, since major defects were not detected in any of the seven cases. Moreover, in most cases the missing features and functionalities needed to construct and complete the standard test survey are ones that could be addressed in the field using simple work-arounds, such as manual entry of ID numbers from barcode tags, or the use of a separate barcode scanner app for collecting barcode data, and the substitution of still images for video records in documenting most routine field observations. Likewise, in the case of missing skip logic features, survey questions could likely be restructured to work around the skip logic functionality missing in selected MSTs. Thus, the application of the evaluation framework appeared to be successful in revealing the relative strengths of particularly well-suited MSTs from a field of acceptable options, something that user opinions and recommendations appeared unable to do (as users’ subjective levels of satisfaction and willingness to recommend MSTs varied little across technology options). Thus, systematic evaluation frameworks such as the one developed in this work may add substantial value in helping implementers select the best available option for their application from among a field of acceptable choices. Moreover, such frameworks may assist MST developers in identifying key areas on which to focus for improving the next version of their products.

### 4.2. Limitations of This Study

Several limitations of this study deserve mention. The MST user survey, which informed the development of the evaluation framework, was taken from a small (*n* = 31) convenience sample consisting primarily of members of a WaSH MST user group that provided relevant responses to an online survey. While the diverse institutional background of respondents and the number of countries represented suggest a plurality of experiences and responses, this sample is by no means representative of all current or potential MST users. Furthermore, it should be noted that few government agencies were included in the sample; this may be indicative of slower adoption of MSTs by government, lower rates of representation on RWSN’s D-group listserve, differences in Internet access or willingness to complete online surveys, or to any number of other factors. Thus, both selection bias and response bias may have been introduced by the convenience sampling approach. Without data on non-respondents within the D-group listserve, as well as on MST users who are not members of the group, it is not possible to assess the extent or nature of these potential biases. The response rate of 4% is also relatively low for online surveys; while it is not possible to determine the reason that this response rate was not higher, we may speculate that factors could include a substantive proportion of inactive members and emails on the D-group list-serve, language barriers among international members, a large proportion of MST nonusers among the listserve members (who may potentially have been more reticent to respond), limited Internet connectivity, limited interest in the survey, or any number of other factors.

Another potential source of bias is the possibility that some respondents may have had relationships or affiliations with the developers of specific MSTs, and conflicts of interest related to such potential relationships cannot be ruled out. Thus, the results obtained from this survey should be considered illustrative, but by no means representative of the attitudes and preferences of all MST users outside of the sample of survey respondents. To the extent that the survey results informed the development of the evaluation framework, these caveats should be kept in mind.

It should also be noted that the results of the evaluation framework are highly sensitive to the STQ used; specifically, the format and content of the questions included in the test questionnaire should be determined with the intended application in mind. Test surveys should be designed to encompass one example of each type of question and/or data type that may be used in the intended monitoring and evaluation applications, and to include multiple different types of skip logic cases that may occur in typical field data collection instruments. The greater the extent to which the STQ can be customized to the intended application, the more representative and useful the evaluation results are likely to be. Furthermore, STQ design should be done with as little prior knowledge as possible of the specific features of individual MSTs to be tested, to avoid the unintentional introduction of bias towards one tool or another. For this study, we attempted to use actual survey questions of the type commonly used in WaSH monitoring and evaluation work, with sufficient diversity in the type of data collected and the structuring of survey questions to highlight the strengths and weaknesses of the different MSTs tested. However, the researchers in this study had some familiarity with the features of several of the candidate MSTs tested, and thus the possibility that this knowledge may have introduced unintentional bias cannot be ruled out.

Furthermore, where MSTs crashed or performed incorrectly during the creation and completion of the STQ by testers, the extent to which these errors are attributable to issues with the MST, the test device, the user, the network, or interactions between these four elements cannot be determined; thus these results reflect performance of the MSTs as deployed with the testers, devices, and networks used.

It should also be noted that the scoring rubric used in this work (equal-weighted rankings across multiple performance categories), while effective in differentiating among the MSTs tested, was very simple, and may not fully reflect the relative priorities different end users place on different aspects and features of WaSH MSTs. For example, the speed and ease of data collection was weighted equally to the speed and ease of form construction in the current study, when for many applications the former (which must be done thousands of times by field workers) may prove far more important than the latter (which may only be done a small number of times by IT professionals). Likewise, cost was weighted equally to performance metrics—when for some small programs cost may be of paramount concern, while for large institutions it may be insignificant relative to performance considerations. Thus, more sophisticated and customizable scoring rubrics may also be able to improve the sensitivity and specificity of this evaluation method for different MST end-users and applications. A sample worksheet providing weighted ranking of the MSTs evaluated in this work is provided for illustrative purposes (Worksheet S1).

Furthermore, it is useful to note that the purpose of this work is primarily to develop and test an MST evaluation method, rather than to rank existing WaSH MSTs. Thus, the sample of MSTs used in this work was neither an exhaustive nor a representative sample, and was selected for illustrative purposes. It would be a mistake to infer that the best-performing MST tool in this study was necessarily the best such tool available at the time the work was performed, or to generalize these results outside of the specific tools and versions tested in the specific time period during which this work was conducted.

Moreover, the testers used in this work were students and staff at UNC, and while they attempted to replicate realistic field conditions and apply the evaluation method with the mindset of field-level WaSH program staff in developing country settings, it is not realistic to assume that the results obtained using this framework for simulated monitoring using the STQ in the US context will be exactly representative of results achieved in the field when MSTs are used by field staff with diverse educational and technical backgrounds in different geographic settings with different questionnaires and different hardware and network service conditions. Thus, the framework is meant to yield results that are indicative, but not necessarily representative, of the typical in-field performance that might be expected of MSTs evaluated for a given application. While the proposed evaluation framework is, to our knowledge, the most rigorous and systematic tool available for assessing MSTs for use in field-level monitoring and evaluation, and may be useful in establishing the overall strengths and weaknesses of different MSTs, implementers are advised to adequately pilot candidate MST(s) under actual field conditions as part of any MST selection, training, or implementation activity.

Finally, it is worth noting that many of the MSTs studied have released updates and/or new versions since the testing activities were conducted; thus, some results and information related to these MSTs may thus already be obsolete. However, the evaluation framework validated in this work, and the performance priorities highlighted by the associated user surveys, are likely to remain useful even as the MSTs tested in this work continue to evolve and mature.

### 4.3. Limitations of MSTs

Some prior studies have seemed to suggest that MSTs can address issues of poor governance, and the lack of sustainability, and/or inadequate post-implementation monitoring of WaSH services [8]. While effective use of MSTs may leverage existing efforts in these areas, it is evidence-based improvement activities, supported by high-quality data collection, which may improve outcomes and sustainability, with or without the use of MSTs. While the capacity of MSTs to improve the quality and efficiency of robust monitoring and evaluation programs may be substantial, MSTs do not inherently solve implementation problems, nor does their use necessarily improve data quality. Without adequate institutional support, training, survey design and implementation expertise, as well as appropriate quality assurance and quality control measures, MSTs may simply facilitate more efficient collection of inaccurate data (i.e., users risk “collecting bad data faster”). In other words, if users ask the wrong questions, advanced apps and smartphones will not improve the quality of the answers. Thus, it is important to view MSTs and other ICTs as one potential class of tools to facilitate a complex process that also includes developing and validating robust questions, operational definitions, and survey instruments, proper sampling, rigorous quality assurance and quality control measures, and effective training, survey implementation and analysis, among others. In combination with these other elements, MSTs can dramatically facilitate the collection of high-quality data to support the implementation of more effective and sustainable programs and activities, and systematic EFs can help implementers select more appropriate MSTs to take advantage of these benefits.

## 5. Conclusions

Based on this work, we conclude that the proposed evaluation framework provides a useful basis for assessing the suitability and relative performance of new and existing MSTs for field-level data collection. As such, it represents the first rigorous MST evaluation framework for such applications, to the best of the authors’ knowledge. The authors note that further customization of the framework and adequate design of appropriate test questionnaires are important for ensuring that future applications of this framework produce results that are as representative as possible of the needs of the intended end-users. Likewise, we conclude that more sophisticated scoring rubrics may improve the sensitivity and specificity of the evaluation method. Finally, while the evaluation results appear to be useful in assessing and comparing promising MST options, end users should adequately pilot candidate MST tools, mobile devices, and networks together in the field as part of any MST selection, training, or implementation activity.

## Figures and Tables

**Table 1:** Quality model for MSTs (Adapted from ISO/IEC 25010 and ISO 9126).

Characteristic	Sub Characteristic	Definition
**Product Modeling**		
Functionality	Suitability	Degree to which an MST meets stated and implied user needs when used under specified conditions
	Accuracy	Degree to which an MST provides accurate results with the needed degree of precision
	Interoperability	Degree to which MSTs can exchange information with other systems and use information that has been exchanged
	Security	Degree to which an MST protects data from unauthorized access by other persons or systems
Reliability	Maturity	Degree to which an MST has overcome initial bugs and defects, and meets needs for reliability under normal operation
	Fault tolerance	Degree to which an MST operates as intended despite the presence of hardware or software faults
	Recoverability (data, process)	Degree to which, in the event of an interruption or a failure, an MST can recover the data directly affected and re-establish the desired state of the system
Usability	Understandability	Degree to which the features and functions of an MST can be understood by users with a wide range of backgrounds and levels of expertise.
	Learnability	Degree to which users with a wide range of backgrounds and levels of expertise can efficiently learn to use an MST to achieve specified goals
	Operability	Degree to which an MST is easy to operate and control
	Attractiveness	Degree to which users perceive an MST’s user interface to be attractive and satisfying to use
Efficiency	Time behavior	Degree to which MST response times, processing times, and throughput rates (when performing functions with adequate hardware and networks) meet or exceed user requirements
	Resource Utilization	Degree to which the amounts and types of resources used by an MST, when performing its functions, meet requirements
Maintainability	Analyzability	Degree of effectiveness and efficiency with which it is possible to assess the impact on an MST of an intended change to one or more of its parts, or to diagnose an MST for deficiencies or causes of failures, or to identify parts to be modified
	Changeability	Degree to which an MST can be effectively and efficiently modified by users without introducing defects or degrading existing product quality
	Stability	Degree to which an MST performs free from failures, interruptions, and unexpected effects
	Testability	Degree of effectiveness and efficiency with which test criteria can be established for an MST and tests can be performed to determine whether those criteria have been met
Portability	Adaptability	Degree to which an MST can effectively and efficiently be adapted for different or evolving hardware, software or other operational or usage environments
	Ease of Installation	Degree of effectiveness and efficiency with which an MST can be successfully installed and/or uninstalled in a specified environment
	Co-existence	The capability of an MST to exist and operate on systems on which other software simultaneously exists and operates
	Replaceability	The capability of an MST to be used in place of another specified MST for the same purpose in the same environment
**Quality-in-Use**		
Effectiveness	User Accomplishment	Accuracy and completeness with which users achieve specified goals
Efficiency	Cost-Benefit	Resources expended in relation to the accuracy and completeness with which users achieve goals
Satisfaction	Usefulness	Degree to which a user is satisfied with their perceived achievement of pragmatic goals, including the results of use and the consequences of use
	Trust	Degree to which a user or other stakeholder has confidence that an MST will behave as intended
	Pleasure	Degree to which a user obtains pleasure from fulfilling their personal needs when using an MST
	Comfort	Degree to which the user is satisfied with his or her physical comfort when using an MST
Freedom from Risk	Economic Risk Mitigation	Degree to which an MST mitigates potential risks to financial status, efficient operation, commercial property, reputation or other resources in the intended contexts of use
	Health and Safety Risk Mitigation	Degree to which an MST mitigates potential risks to people in the intended contexts of use
	Environmental Risk Mitigation	Degree to which an MST mitigates potential risks to property or the environment in the intended contexts of use
Context Coverage	Context Completeness	Degree to which an MST can be used with effectiveness, efficiency, freedom from risk and satisfaction in all the specified contexts of use
	Flexibility	Degree to which an MST can be used with effectiveness, efficiency, freedom from risk and satisfaction in contexts beyond those initially specified in the requirements

**Table 2:** MSTs used by survey respondents.

MST	Frequency: *n* (%)
ODK	9 (29%)
Proprietary/Custom MST	7 (23%)
FLOW	4 (13%)
Magpi	1 (3%)
Iformbuilder	1 (3%)
Viewworld	1 (3%)
Mobiles4Water	1 (3%)
Other	7 (23%)

*n* = 31.

**Table 3:** MST applications.

Response Category	Frequency: *n* (%)
Waterpoint Data Collection	24 (75%)
Community surveys	21 (66%)
Household Surveys	19 (59%)
Waterpoint Mapping	18 (56%)
Field activity reporting	18 (56%)
Sanitation data collection	13 (41%)
WaSH committee surveys	10 (31%)
Sanitation Mapping	9 (28%)
Other	11 (34%)
Well-drilling data collection	2 (6%)
Monitoring water treatment plant performance	1 (3%)
Water meter readings	1 (3%)
Chlorine delivery reporting	1 (3%)
School and clinic WaSH monitoring	(3%)

*n* = 31 (Respondents could select more than one option).

**Table 4:** Reason for selecting current MST.

Response Category	Frequency: *n* (%)
Ease of data input	19 (61%)
Ability to export data into desired format	16 (52%)
Cost	15 (48%)
Ease of survey creation	13 (42%)
Intuitive navigation and functionality	10 (32%)
Compatibility with existing hardware and software	10 (32%)
Auto-upload of data when networks are available	9 (29%)
Quality and availability of user support	9 (29%)
Recommendation from another user	7 (23%)
Attractive user interface	6 (19%)
Logical form submission process	5 (16%)
Speed of data analysis and reporting features	5 (16%)
Other (please specify)	5 (16%)
Extent of adoption of tool by other organizations	4 (13%)
Ability to try MST before committing	3 (10%)
Privacy and security of data	1 (3%)
Speed of uploads	1 (3%)

*n* = 31 (Respondents could select more than one option).

**Table 5:** Mean level of satisfaction with current MST: score out of 10 (standard deviation).

Satisfaction with Field Data Collection	8.0 (1.3)
Satisfaction with dashboard	5.6 (1.9)
Would recommend	28 (90%)

*n* = 31.

**Table 6:** Mean level of satisfaction by MST used: score out of 10 (standard deviation).

MST	Satisfaction with App	Satisfaction with Dashboard	Would Recommend	*n*
ODK	8.6 (0.9)	4.3 (1.5)	100%	9
FLOW	8.3 (1.3)	7.0 (2.6)	100%	4
Proprietary	7.9 (1.2)	7.0 (1.8)	100%	7
Other	7.5 (1.5)	5.2 (0.9)	73%	11

**Table 7:** Most beneficial MST features.

Response Category	Frequency: *n* (%)
Ease of use: general	6 (19%)
GPS features	6 (19%)
Flexibility	4 (13%)
Speed/Efficiency	4 (13%)
Ease of uploads	4 (13%)
Reliability	3 (10%)
Ease of use: survey creation	3 (10%)
Compatibility with device of choice	3 (10%)
Skip logic	2 (6%)
Ease of use: input	2 (6%)
Cost	2 (6%)
Photo/video capture	2 (6%)
Avoid manual data entry	2 (6%)
SMS features	1 (3%)
Trialability	1 (3%)

*n* = 31 (Respondents could select more than one option).

**Table 8:** Most problematic MST features.

Response Category	Frequency: *n* (%)
GPS	4 (13%)
Output and Reporting	4 (13%)
Unavailable on desired devices	2 (6%)
Data loss	2 (6%)
User interface (data collection)	1 (3%)
Screen resolution (hard to read in bright light)	1 (3%)
Lack of SMS capabilities	1 (3%)
Inability to view GPS coordinates on a map	1 (3%)

*n* = 31.

**Table 9:** Main benefits of dashboard.

Response Category	Frequency: *n* (%)
Ease of use	6 (19%)
Export options	6 (19%)
Map features	2 (6%)
Organization	1 (3%)

*n* = 31.

**Table 10:** Most important dashboard features.

Response Category	Frequency: *n* (%)
Reporting	5 (16%)
Performance over slow connections	3 (9%)
Online data management	2 (6%)
Additional support	1 (3%)
Data visualization	1 (3%)
Maps	1 (3%)
Supported languages	1 (3%)

*n* = 31.

**Table 11:** General characteristics of MSTs tested (as of 2015).

Parameter	FLOW	ODK	Magpi	iFormbuilder	Fulcrum	Mwater	Survey CTO
1.1 Mobile platform compatibility	Android	Android	Android; iOS; Nokia	iOS	Android; iOS	Android; iOS	Android
1.2 Mobile platforms tested	Android	Android	Android; iOS	iOS	Android; iOS	Android; iOS	Android
1.3 Mobile devices tested	Samsung Galaxy S II Skyrocket; Samsung Galaxy Stellar; Huawei Impulse	Samsung Galaxy S II Skyrocket; Huawei Impulse	Samsung Galaxy S II Skyrocket; Samsung Galaxy Stellar; iPhone 5	iPhone 4s; iPhone 5; iPhone 5s	Samsung Galaxy S II Skyrocket; Samsung Galaxy Stellar; iPhone 5; iPhone 5s	Samsung Galaxy S II Skyrocket; Motorola Droid Mini; iPhone 5	Samsung Galaxy S II Skyrocket; Motorola Droid Mini
1.4 Web browsers used to test dashboard	Chrome; Firefox; Internet Explorer	Chrome; Firefox; Safari	Chrome; Firefox; Safari	Chrome; Firefox; Safari	Chrome; Firefox; Safari	Chrome; Firefox; Safari	Chrome; Firefox; Safari
1.5 OS used to test dashboard	Windows 7; Mac OS X	Windows 7; Mac OS X	Windows 7; Mac OS X	Windows 7; Mac OS X	Windows 7; Mac OS X	Windows 7; Mac OS X	Windows 7; Mac OS X
1.6 Does app function offline?	Yes	Yes	Yes	Yes	Yes	Yes	Yes
1.7 Does Dashboard function offline?	No	No	No	No	No	No	No
1.8 Cost of MST (USD)	Variable; approx. $10k for one instance with setup and training	Free	$5k/year for 10 k uploads; $10k/year for 20 k uploads	Smart Enterprise: $100; Exploring $2k; Growing $5k; Emerging $10k	Variable: $29/month (1 user); $99/month (5 users); $399/month (25 users); $749/month (50 users)	Free	Variable: $99/month (10 users); $399/month (unlimited users).
Est. cost for 10 users and 10k uploads over 1 year	$10,000	0	$5,000	$5,000	$4,788	0	$1,188
Cost Rank	4	1	3	3	3	1	2

**Table 12:** Performance and features.

Parameter	FLOW	ODK	Magpi	iFormbuilder	Fulcrum	Mwater	Survey CTO
App							
5.14a Did app crash?	0/4	1/4	0/4	1/4	0/4	0/3	0/3
5.14b Which features caused app to crash?	N/A	loading form, GPS	N/A	Saving form	N/A	N/A	N/A
5.16a App functions missing?	No	Yes	Yes	Yes	Yes	No	No
5.16b Which functions missing?	N/A	Barcode scanner	Barcode scanner, video	Barcode scanner	Barcode scanner, video	N/A	N/A
5.17a Did any features perform incorrectly?	2/4	1/4	1/4	1/4	0/3	2/3	0/3
5.17b Which features performed incorrectly?	GPS, video	Open form; submit form; GPS	Loading survey	GPS	N/A	GPS, video	N/A
Dashboard							
6.18a Did any dashboard functions perform incorrectly?	Yes	No	No	Yes	No	No	No
6.18b Which dashboard functions performed incorrectly?	Skip logic	N/A	N/A	Skip logic	N/A	N/A	N/A
Overall							
8.1 Were any data points not received?	No	Yes	No	No	No	No	Yes
Performance Score (0 = perfect score)	1.5	1.5	1.25	2.5	1	1.75	0
Performance Rank	4	4	3	7	2	6	1

*n* = 4.

**Table 13:** Ease of Use.

Parameter	FLOW	ODK	Magpi	iFormbuilder	Fulcrum	Mwater	Survey CTO
App: Level of Difficulty (Likert Scale [1–5]: 1 = little difficulty; 5 = very difficult)							
2.2 App installation	2.67	3.33	2	2	1.67	1	3
2.2 App configuration	3	4.25	2	2	1.75	2.5	2.5
3.2 Account setup	1.67	2.5	1	1.75	1	1	1.67
5.1 App navigation	2	2	2	2	1.5	2.33	2
Dashboard: Level of Difficulty (Likert Scale [1–5]: 1 = little difficulty; 5 = very difficult)							
6.1 Dashboard navigation	1.75	2.5	3	2.5	1.5	1.33	2.33
6.3 Form construction	2.25	3	3.25	2.75	1.75	2	4.67
Overall Level of Difficulty (Sum of above level-of-difficulty scores (6 = best possible score; 30 = worst possible score))							
Overall Ease of use Score	13.33	17.58	13.25	13	9.17	10.75	15
Overall Ease of use Rank	5	7	4	3	1	2	6

*n* = 4.

**Table 14:** Learning curve (estimated hours to learn to proficiently use MST (average of four raters)).

Parameter	FLOW	ODK	Magpi	iFormbuilder	Fulcrum	Mwater	Survey CTO
App							
5.3 Training to use app	0.7	0.7	0.7	0.7	0.7	0.6	0.6
Dashboard							
6.7 Training to use dashboard	1.9	5	1.9	1.9	1.4	1.4	3.5
Overall							
Training to use app & dashboard	2.6	5.7	2.6	2.6	2.1	2.0	4.1
Learning curve Rank	3	7	3	3	2	1	6

*n* = 4.

**Table 15:** Speed.

Parameter	FLOW	ODK	Magpi	iFormbuilder	Fulcrum	Mwater	Survey CTO
App							
5.8 Adjusted survey completion time (average mins)	2.3	2.0	2.4	1.7	2.2	3.2	2.7
5.9a One or more app functions are slow	0.67	0.75	0.33	0.33	0	0.67	0.33
5.9b Slow app functions	Video, GPS	Video, GPS, submission	GPS	GPS	None	GPS, photo	GPS
Dashboard							
6.9 Adjusted survey creation time	19.1	26.3	32.9	28.8	20.1	22.6	29.1
6.10a One or more dashboard functions slow	0.67	0	0.67	0	0	0	0.5
6.10b Slow dashboard functions	Saving questions, exporting reports, exporting data, switching tabs	None	Saving, creating new questions	None	None	None	Programming survey offline
Overall							
Overall speed score (lower score = faster)	17	13	21	9	7	17	19
Speed Rank	4	3	7	2	1	4	6

*n* = 4.

**Table 16:** Equal-weighted composite score and overall rank (lowest composite score is best).

Parameter	FLOW	ODK	Magpi	iFormbuilder	Fulcrum	Mwater	Survey CTO
Overall							
Composite Score	20	22	20	18	9	14	21
Overall Rank	4	7	4	3	1	2	6

## Supplementary Materials

The following are available online at www.mdpi.com/1660-4601/13/9/840/s1, Figure S1: MST User Survey, Figure S2: Data Management Value Chain, Table S1: MST Evaluation Questionnaire, Table S2: Standard Evaluation Survey, Table S3: Detailed Definitions of Survey Applications, Worksheet S1: Calculator for weighted ranking of tested MSTs.
