# Supplementary material for: Evaluating Mobile Survey Tools (MSTs) for Field-Level Monitoring and Data Collection: Development of a Novel Evaluation Framework, and Application to MSTs for Rural Water and Sanitation Monitoring
Source: Int J Environ Res Public Health. 2016 Aug 23;13(9):840. doi: 10.3390/ijerph13090840 (PMC5036673; doi:10.3390/ijerph13090840)
Supplement: Supplementary file 1 [file ijerph-13-00840-s001.pdf]

Supplementary Materials: Evaluating Mobile Survey Tools (MSTs) for Field-Level Monitoring and Data Collection: Development of a Novel Evaluation Framework, and Application to MSTs for Rural Water and Sanitation Monitoring

Michael B. Fisher, Benjamin H. Mann, Ryan D. Cronk, Katherine F. Shields, Tori L. Klug and Rohit Ramaswamy

ICTs in WaSH Evaluation

This survey focuses on information and communication technologies (ICTs) for mobile data collection and analysis in Water Sanitation, and Hygiene (WaSH) projects. It is designed to assess user needs in selecting ICT tools and their experiences using them in the field. Please answer the following questions about your organization and its use of ICTs for WaSH-related projects. If your organization does not currently use or has not used ICTs in the past, we kindly ask that you do not participate in this survey.

1. What organization do you currently work with?

Organization Name:

Approximate number of employees:

***2. What is the primary WaSH ICT tool your organization uses for mobile data collection and analysis? ("WaSH ICT tool" refers to the software or app that you are using, not the mobile phone or other hardware that you use to collect data)**

- FLOW
- Magpi
- iFormBuilder
- mWater
- PoiMapper
- Open Data Kit
- Kobe
- Fulcrum
- ArcGIS Mobile
- None
- Other (please specify)

3. What does your organization use this tool for? (Mark all that apply)

- Community surveys
- WaSH committee surveys
- Waterpoint mapping
- Waterpoint data collection
- Sanitation mapping
- Household surveys
- Field activity reporting
- Sanitation facility data collection
- Other (please specify)

ICTs in WaSH Evaluation

4. Select up to 5 characteristics that you considered most important when you selected your tool.

- [ ] Recommendation from another user
- [ ] Cost
- [ ] Ease of survey creation
- [ ] Ability to export data into desired format
- [ ] Compatibility with existing hardware and software
- [ ] Auto-upload of data when networks are available
- [ ] Privacy and security of data
- [ ] Extent of adoption of tool by other organizations
- [ ] Intuitive navigation and functionality
- [ ] Attractive user interface
- [ ] Ease of data input
- [ ] Logical form submission process
- [ ] Speed of uploads
- [ ] Speed of data analysis and reporting features
- [ ] Ability to try ICT before committing
- [ ] Quality and availability of user support
- [ ] Other (please specify)

Most ICT tools have two primary components: a field data collection system and a web-based dashboard (for survey and data management). Please answer the following questions about each component of the tool your organization has used:

***5. Rate your satisfaction with the tool's performance for field data collection: (1=very unsatisfied, 10=very satisfied)**

1	2	3	4	5	6	7	8	9	10
○	○	○	○	○	○	○	○	○	○

***6. Please list up to 3 features of the tool's performance in the field that you found most beneficial to your work.**

7. Why were these features beneficial ?

***8. Please list up to 3 features of the tool's performance in the field that you found problematic.**

9. Why were these features problematic ?

ICTs in WaSH Evaluation

***10. Rate your satisfaction with the tool's online dashboard for analysis and reporting: (1=very unsatisfied, 10=very satisfied)**

1	2	3	4	5	6	7	8	9	10
○	○	○	○	○	○	○	○	○	○

***11. Please list up to 3 features of the tool's online dashboard that you found most beneficial to your work.**

12. Why were these features beneficial ?

***13. Please list up to 3 features of the tools' online dashboard that you found problematic.**

14. Why were these features problematic ?

***15. Would you recommend this tool to another organization looking for an ICT solution?**

○ Yes

○ No

Reason?

16. Is there anything else you would like to share with us about your ICT tool?

Figure S1. MST User Survey.

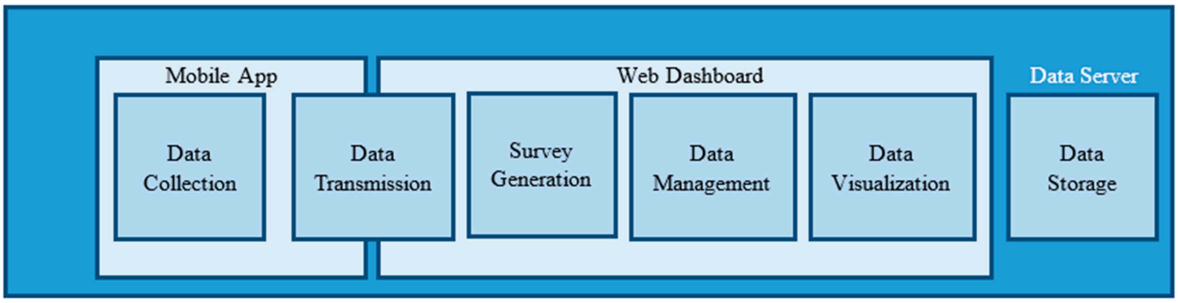

Figure S2. Data Management Value Chain.

Table S1. MST Evaluation Questionnaire.

Evaluator Name: ____________________
Date: ____________________
MST: ____________________
Version No.: ____________________
Developer: ____________________

UNC WATER INSTITUTE

1. General (Pre-Evaluation)		
1.1 What mobile platforms does the app run on?		
1.2 What mobile platform are you testing the app on?		Version:
1.3 What model phone are you testing the app on?		
1.4 What browser are you testing the dashboard on?		
1.5 Which operating system (OS) are you testing the dashboard on?		
1.6 Does the app function offline?	Yes ☐ No ☐	
1.7 Does the dashboard function offline?	Yes ☐ No ☐	
1.8 Cost of the tool:		
Setup		
2. Mobile Application (App)		
Findability	2.1 Where can you access the app?	App Store ☐ Play Store ☐<br>Developer's Website ☐<br>Other ☐
Installation	2.2 How difficult is it for a new user to install the app on the mobile device?	Likert scale (1–5)
Configuration	2.3 How difficult is it for new users to independently configure the app to begin data collection?	Likert scale (1–5)
Security	2.4 Is a login required to access the app?	Yes ☐ No ☐
Form Retrieval	2.5 What steps are required to retrieve forms from the app?	
Language	2.6. What language(s) does the app operate in?	
3. Online Dashboard		
Security	3.1 Is a login required to access the dashboard?	Yes ☐ No ☐
Configuration	3.2 Level of difficulty to set up a new account	Choose an item.
Software Install (optional)	3.3 Is any software installation required?	Yes ☐ No ☐<br>If yes, which?____________
Language	3.4 What language(s) does the dashboard operate in?	
4. User Interface (UI)		

	4.1 Color contrast (Legibility)	Likert scale (1–5)
	4.2 Typography (Legibility)	Likert scale (1–5)
	4.3 Icons (Ease of understanding)	Likert scale (1–5)
Field Use		
5. Mobile App		
Navigation	5.1 How difficult is it to navigate the layout of the app?	Likert scale (1–5)
Information Architecture	5.2 Are the functions of the app organized intuitively?	Yes ☐ No ☐
Training	5.3 What amount of training is required for users to operate the app, if any?	None<br><30 min<br>30–59 min<br>1–4 h<br>4–8 h<br>>8 h
	5.4 What types of training materials are available, if any?	Downloadable materials ☐<br>Video ☐ In Person Training ☐<br>Other ☐
Transmission	5.5 How are surveys updated on the app?	
	5.6 What methods of data submission are available?	
Translation	5.7 Are all translated survey languages available on the app?	
Efficiency	5.8a What is the amount of time taken to complete the test survey?	______min
	5.8b What is the adjusted amount of time taken to complete the test survey (adjusting for any questions that could not be completed due to missing features)?	______min
	5.9a Did any functions of the app operate slowly?	Yes ☐ No ☐
	5.9b What functions of the app operate slowly, if any?	
Interoperability	5.10 Can multiple users complete surveys on a single device?	Yes ☐ No ☐
Resource Utilization	5.11 What amount of battery drain does the app use?	
Code Visibility	5.12 Is the code for the app viewable to average users?	Yes ☐ No ☐
Code Editability	5.13 Is the code for the app changeable to average users?	Yes ☐ No ☐
Risk of Data Loss	5.14a Did the app crash?	Yes ☐ No ☐
	5.14b Which features caused the app to crash?	
Trialability	5.15 Can users pilot test the app with a free trial?	Yes ☐ No ☐
Functional adequacy	5.16a Were any functions missing that were required to complete the test survey?	Yes ☐ No ☐
	5.16b Which functions were missing that were required to complete the test survey, if any?	
Functional Correctness	5.17a Did any functions perform incorrectly during the test survey?	Yes ☐ No ☐

	5.17b What functions performed incorrectly, if any?	
6. Online Dashboard- Actions: Build survey, publish survey, modify survey, send survey to mobile devices.		
Navigation	6.1 How difficult is it to navigate the dashboard?	Likert scale (1–5)
Dashboard Layout	6.2 Are the functions of the dashboard organized intuitively?	Yes ☐ No ☐
Form Generation	6.3 How difficult is it to construct a form?	Likert scale (1–5)
	6.4 What survey question types can be generated?	
	6.5 Can skip logic be included?	Yes ☐ No ☐
Transmission	6.6 How are surveys distributed to mobile devices?	
Training	6.7 What level of training is required for new users to independently operate the dashboard?	None<br><30 min<br>30–59 min<br>1–4 h<br>4–8 h<br>>8 h
	6.8 What types of training materials are available, if any?	Downloadable material ☐<br>Video ☐<br>In Person Training ☐<br>Other ☐
Efficiency	6.9 What is the amount of time taken to construct the test form?	_____min
	6.9 What is the adjusted amount of time taken to construct the test form (adjusting for any items that could not be created due to missing features)?	_____min
	6.10a Did any functions of the dashboard operate slowly?	Yes ☐ No ☐
	6.10b What functions of the dashboard operate slowly, if any?	
Translation	6.11 What language translation functions exist in the dashboard, if any?	
Resource Utilization	6.12 What amount of RAM usage is required to use the dashboard?	
Code Visibility	6.13 Is the code for the dashboard viewable to average users?	Yes ☐ No ☐
Code Editability	6.14 Is the code for the dashboard changeable to average users?	Yes ☐ No ☐
Risk of Data Loss	6.15a Did the dashboard crash?	Yes ☐ No ☐
	6.15b What features caused the dashboard to crash?	
Trialability	6.16 Can users pilot test the dashboard for free?	Yes ☐ No ☐
Functional adequacy	6.17a Where any functions missing that were needed to complete the test form?	Yes ☐ No ☐
	6.17 Which functions were missing that were needed to complete the test form, if any?	
Functional Correctness	6.18a Did any functions perform incorrectly?	Yes ☐ No ☐
	6.18 What functions performed incorrectly, if any?	

Transmission	6.19 Once the form is completed, how difficult is it to send the survey to the mobile device?	Likert scale (1–5)
Data Management		
7. Mobile App		
Data Management	7.1 Can data points be edited in the app?	Yes ☐ No ☐
	7.2 Can data points be reviewed in the app?	Yes ☐ No ☐
	7.3 Can survey questions be edited in the app?	Yes ☐ No ☐
Data Transmission	7.4 What process is used to verify and/or review data submission?	
8. Online Dashboard		
Data Transmission	8.1 How many data points were not received correctly from the app?	
Data Management	8.2 Which items, if any, can be edited?	
	8.3 What formats can data be exported in?	.txt ☐ .csv ☐ .xls ☐ other ☐
Data Reporting	8.4 What type of reports can be generated?	
Data Visualization	8.5 What type of visualizations can be generated?	
Data Storage	8.6 What format is data stored online?	
	8.7 What amount of storage is provided?	
Data Security	8.8 What security features exist in the dashboard to safeguard data?	
9. Additional Information		
User-Developer Relations	9.1 What level of support exists from the developer for MST users?	
User Community Size	9.2 How many users/groups use the MST?	
User Community Support	9.3 Is there an active user community for the MST?	Yes ☐ No ☐
Updates	9.4 How frequently are updates released?	
Bug Fixes	9.5 What is the process for fixing bugs?	
	9.6 How long does it take to fix bugs?	

Table S2. Standard Test Questionnaire.

Question	Reponses
**Water Point Name:**	[Free text]
**Date**	[Date]
**Type of Source**	(1) Piped water into dwelling; (2) Piped water to yard/plot; (3) Public tap/standpipe; (4) Borehole with manual pump; (5) Protected dug well; (6) Unprotected dug well; (7) Protected spring; (8) Unprotected spring; (9) Rainwater collection; (10) Bottled, sachet, or "pure water" water; (11) Cart with small tank/drum; (12) Tanker-truck; (13) Surface water (river, dam, lake, pond, stream, canal, irrigation channels); (14) other (specify); (−333) Not applicable; (−444) Don't know; (−555) Decline to state
**Location**	[GPS coordinates]
**Functional?**	Y/N
**Flow Rate (L/m)**	Dependent on Functionality

**If flow rate < 10, why?**	Dependent on Flow rate < 10; (1) pump inadequate; (2) slow recharge; (3) other; (777) Not applicable; (888) Don't know; (999) Decline to state
**Photo of Source**	[Image]
**Water Quality Sample collected?**	Dependent on Functionality
**WQ ID**	Dependent on WQ sample
**Video of functionality**	[Video]
**How many strokes to get water**	Dependent on Type = borehole with manual pump and Functionality = yes
**Water quality description (clear, turbid, etc.)**	(1) Clear; (2) turbid; (3) cloudy; (4) black; (5) red; (6) yellow; (7) white; (8) green; (9) Oily; (10) Other (describe)

Table S3. Detailed Definitions of Survey Applications.

Term	Definition
**Waterpoint Data Collection**	Collecting data about the characteristics of water sources, (e.g., type, location, functionality, water quality, reliability, etc.)
**Community surveys**	Collecting data about water and sanitation services at the community level (e.g., types and adequacy of services present in the community, community management structure and functionality, etc.)
**Household Surveys**	Collecting data about water and sanitation services at the household level (e.g., types and adequacy of services used by the household, etc.)
**Waterpoint Mapping**	Collecting data about the existence of waterpoints, such as type and location (but not about characteristics such as functionality, water quality, reliability, etc.)
**Field activity reporting**	Reporting on outputs and activities conducted by field staff (trainings conducted, facilities constructed, etc.)
**Sanitation data collection**	Collecting data about the characteristics of sanitation facilities (e.g., type, location, functionality, condition, service quality, reliability, etc.)
**WaSH committee surveys**	Collecting information about WaSH committee presence, functionality, activities, and composition, etc.
**Sanitation Mapping**	Collecting data about the existence of sanitation facilities, such as type and location (but not about characteristics such as functionality, conduction, service quality, reliability, etc.)
**Other:**	
**Well-drilling data collection**	User-entered; no definition provided
**Monitoring water treatment plant performance**	User-entered; no definition provided
**Water meter readings**	User-entered; no definition provided
**Chlorine delivery reporting**	User-entered; no definition provided
**School and clinic WaSH monitoring**	User-entered; no definition provided
